# The impact of organizational blindness on nurses’ commitment in healthcare settings

**DOI:** 10.1108/JHOM-08-2025-0488

**Published:** 2026-01-07

**Authors:** Seda Berkay, Seda Tuğba Baykara Mat

**Affiliations:** Nursing Department, Istanbul Okan University, İstanbul, Turkey; Nursing Department, Istanbul Beykent University – Beylikduzu Campus, Istanbul, Turkey

**Keywords:** Organizational commitment, Organizational blindness, Nursing workforce, Human resource management, Patient safety

## Abstract

**Purpose:**

This study investigates the relationship between organizational blindness and organizational commitment among nurses, exploring how demographic and professional factors shape affective, normative and continuance commitment. By emphasizing workforce well-being, organizational transparency and sustainable healthcare management, the study supports the United Nations Sustainable Development Goals of Decent Work and Economic Growth.

**Design/methodology/approach:**

A cross-sectional design was used with 269 nurses employed in a private hospital in Türkiye. Data were collected using a Demographic Information Form, the Organizational Commitment Scale and the Organizational Blindness Scale. Descriptive statistics, *t*-tests, ANOVA, Pearson correlation, linear regression and logistic regression were conducted. Assumptions of normality, homoscedasticity, autocorrelation, and outlier independence were confirmed.

**Findings:**

Nurses reported moderate levels of organizational blindness and commitment. Blindness was significantly and negatively but low correlated with commitment (r = −0.266, *p* < 0.001), explaining 7.1% of the variance (*R*^2^ = 0.071). Being married (OR = 2.05, *p* = 0.031) and having longer professional experience (*p* = 0.045) predicted higher commitment, whereas male gender and rotating shifts were linked to greater blindness.

**Research limitations/implications:**

The single-site, cross-sectional design limits causal inference and generalizability. Future multi-center and longitudinal studies are recommended.

**Practical implications:**

Healthcare leaders should promote open communication, fair scheduling, mentorship and professional development to enhance commitment and reduce blindness.

**Social implications:**

Addressing organizational blindness and strengthening commitment can improve nurse retention, organizational culture and patient care quality.

**Originality/value:**

A focused literature search (PubMed, Scopus and Web of Science; 2000–2025) revealed no prior Turkish empirical study on this link.

## Introduction

In today's fast-paced work environment, employee well-being is a key determinant of organizational success, retention, and commitment. Employing qualified professionals and ensuring their long-term engagement enhances performance and provides a competitive advantage. Within this context, organizational commitment is a cornerstone of organizational sustainability, reflecting the alignment between employees’ personal goals and organizational values (Büyükbeşe and Aslan, 2019). Employees with strong commitment tend to contribute more actively, foster positive workplace relationships, and exhibit lower turnover rates (Şahan, 2020).

In healthcare—an inherently dynamic sector shaped by continual scientific and technological change—nurses’ organizational commitment plays a vital role in maintaining care quality, improving hospital performance, and strengthening public trust (Chang, 2014) .

However, employees who remain in the same roles for extended periods may become overly accustomed to routines, develop a preference for stability, and resist change. Such resistance may diminish their capacity to recognize emerging opportunities or risks and, over time, lead to organizational blindness—a metaphorical condition where individuals or institutions fail to perceive internal inefficiencies or respond to critical issues (Fronzetti Colladon *et al*., 2023; Acar and Mete, 2023). As organizations become more rule-bound and less responsive to their environments, the likelihood of organizational blindness increases (Balarezo *et al*., 2024). In contrast, those that promote reflection, adaptability, and open communication are better positioned to sustain performance and resilience.

Although the literature on organizational commitment is extensive, few studies directly investigate its relationship with organizational blindness, particularly in the nursing context. Existing research has largely focused on predictors such as leadership trust, ethical climate, job satisfaction, and organizational citizenship behavior (Ivziku *et al.*, 2024; Khanian *et al.*, 2024). Within healthcare management scholarship, organizational blindness has been examined in different forms—most often as strategic or ethical blindness (Fotaki, 2015; American College of Healthcare Executives [ACHE], 2014)—or discussed in relation to leader–member exchange and knowledge-sharing mechanisms (Acar and Mete, 2022). Fotaki and Hyde (2015) conceptualized “organizational blind spots” as psychological and structural mechanisms of splitting, blame, and idealization that obscure systemic problems within healthcare institutions. Similarly, Catino (2013) and Seymen *et al*. (2016) described organizational myopia as a failure to learn or adapt caused by entrenched routines, cognitive biases, and hierarchical rigidity.

Despite the growing relevance of these concepts, empirical studies connecting organizational blindness and commitment among nurses remain limited, especially within the Turkish context. This gap is significant given that unrecognized inefficiencies and communication barriers can weaken nurses' emotional engagement, reduce accountability, and threaten patient safety. Understanding how blindness relates to commitment can therefore offer valuable insights for improving leadership, transparency, and workforce sustainability in healthcare institutions.

To ensure a systematic approach, a focused literature search was conducted to identify studies addressing the link between organizational blindness (and related constructs such as willful blindness or organizational myopia) and commitment in nursing and healthcare. Searches were carried out in PubMed, Scopus, and Web of Science databases, covering the years 2000–2025, using the following keywords: “organizational blindness,” “willful blindness,” “organizational myopia,” “organizational commitment,” “nursing,” and “healthcare professionals.” This review confirmed that, while organizational commitment has been extensively explored, no prior Turkish study has empirically examined its association with organizational blindness.

Accordingly, the present study aims to fill this gap by examining the relationship between organizational blindness and organizational commitment among nurses working in hospital settings. By focusing on demographic and professional factors, this research also seeks to clarify which subgroups may be most vulnerable to reduced commitment due to organizational blindness. The study's findings are expected to contribute to the development of evidence-based strategies for healthcare leaders to enhance transparency, strengthen workforce engagement, and promote sustainable organizational performance.

### Organizational blindness and commitment

Organizational blindness refers to the failure of an organization or its members to recognize internal inefficiencies, risks, or opportunities for improvement. It arises from habitual behaviors, resistance to change, and cognitive biases that hinder objective evaluation (Balarezo *et al*., 2024; Fasolo *et al*., 2024). The concept aligns with bounded rationality, which suggests that individuals and organizations make decisions within cognitive and informational limits (Simon, 2016). Over time, these limitations create “blind spots” that obscure critical processes and hinder performance (Lumineau and Oliveira, 2018).

Blindness is reinforced by rigid hierarchies, routine-based task systems, and weak feedback mechanisms that discourage critical reflection (Weick and Sutcliffe, 2015). It differs from organizational silence, which involves the deliberate withholding of concerns, and from burnout or change fatigue, which stem from emotional exhaustion. Instead, blindness emphasizes a collective lack of awareness or misperception. In healthcare, it may manifest as unrecognized workload imbalances, unsafe practices, or communication breakdowns that compromise patient outcomes (Cleary and Duke, 2019).

At a structural level, organizational blindness is sustained by poor information flow and limited interaction with the external environment. When institutions fail to exchange knowledge or reflect on environmental feedback, they risk overlooking emerging threats and losing strategic agility (Fronzetti Colladon *et al*., 2023). Culturally, it often coexists with low psychological safety and defensive routines where mistakes are hidden rather than analyzed (Edmondson, 1999).

Nurses are particularly vulnerable to this phenomenon. Constant exposure to heavy workloads, ethical dilemmas, and emotional labor can narrow situational awareness and normalize unsafe routines (Cleary and Duke, 2019). Low wages, limited career development, and prolonged work under the same supervision further erode commitment and competence (Seymen *et al*., 2016). Such conditions contribute to communication failures, interpersonal conflicts, and workforce instability, all of which threaten organizational sustainability (Balarezo *et al*., 2024).

In contrast, organizations that cultivate a strong safety culture and open communication can prevent blindness by promoting psychological safety, reflective dialog, and cross-unit learning (Weick and Sutcliffe, 2015). Heffernan (2011) describes this phenomenon as willful blindness—the conscious neglect of visible risks or ethical concerns. Related frameworks, such as Vaughan's (2005) “normalization of deviance” and Dekker's (2011) “drift into failure,” explain how unsafe practices gradually become normalized within complex systems.

In healthcare, this may appear as underreporting of errors, acceptance of chronic understaffing, and declining psychological safety (Banja, 2010; Sexton *et al.*, 2021). Such patterns directly threaten nurses’ engagement and commitment. When leaders fail to recognize systemic issues, perceptions of neglect and inequity arise, weakening affective and normative commitment (Hult *et al*., 2023).

Conversely, strong organizational commitment can mitigate the effects of blindness. Committed nurses are more likely to voice concerns, initiate improvements, and advocate for safer practices (Tucker *et al*., 2002). Drawing on safety culture theory, voice and silence literature (Edmondson, 1999), and the Job Demands–Resources model, this study conceptualizes organizational blindness as a contextual stressor that diminishes belonging and engagement.

Ultimately, the interplay between blindness and commitment is pivotal for healthcare performance. Commitment promotes positive behaviors and continuity, while blindness undermines adaptability and learning. Encouraging reflective leadership, open dialog, and continuous learning can reduce blindness and reinforce commitment, thereby supporting sustainable and high-quality healthcare delivery. This study contributes to the literature by empirically examining this relationship among hospital nurses and offering actionable insights for strengthening organizational transparency and workforce resilience.

### Hypotheses development

Based on the literature review, the following hypotheses were proposed.

H1.
Among nurses, organizational blindness is negatively associated with affective commitment.

H2.
Among nurses, organizational blindness is negatively associated with normative commitment.

H3.
Organizational blindness is negatively associated with continuance commitment among nurses.

## Methods

### Study design

This study adopted a cross-sectional, exploratory, and correlational design in accordance with the STROBE (Strengthening the Reporting of Observational Studies in Epidemiology) guidelines (von Elm *et al*., 2007). This design was chosen to analyze existing conditions and to examine the relationships among variables. A cross-sectional and correlational approach provided a solid framework for capturing a snapshot of the current situation, gaining a deeper understanding of variable interactions, and broadening the scope of the investigation.

### Settings

The study was conducted in a private hospital located in İzmit, Kocaeli, Türkiye. The hospital was selected due to its performance-based evaluation system, education-oriented institutional culture, and strong emphasis on supporting nurses’ professional development, tailored to their individual interests and competencies. The organization regularly identifies nurses’ developmental needs through performance appraisals and provides in-service training programs accordingly. In addition, the institution encourages nurses’ participation in scientific activities and offers psychological support mechanisms to enhance staff well-being.

The hospital operates on a 12-h shift system without routine rotations. The absence of rotation allows nurses to gain long-term experience within specific units, promoting continuity in team relationships. However, this structure may also contribute to the routinization of tasks, creating conditions conducive to organizational blindness. Moreover, extended shifts may influence both workload and fatigue levels, potentially shaping nurses’ perceptions of organizational commitment and blindness. Therefore, the unique characteristics of this setting should be carefully considered when interpreting the findings.

The nurses at the hospital participate in a structured orientation program before starting their duties. While this process facilitates their adaptation to the organizational culture, it may also influence how they form emotional attachment to the organization. Because these institutional characteristics have the potential to shape the results, the generalizability of the findings is limited. The study may provide valuable insights for private hospitals with similar structural features, but the results should not be directly generalized to public hospitals or healthcare institutions with rotation systems.

### Study population and sampling

The study population consisted of 280 nurses employed at a private hospital in İzmit, Kocaeli, Türkiye, during the research period. The study was conducted with near-census coverage. Nurses who were actively working at the time of data collection, voluntarily agreed to participate, held a nursing position, and were not on leave or sick leave were included in the study.

The sample size was calculated using the known population formula with a 95% confidence level and a 5% margin of error, indicating that a minimum of 162 nurses was required for the study. To minimize data loss, the researchers aimed to reach at least 200 participants, and ultimately 269 nurses were included in the analysis (96% response rate). This high participation rate strengthens the internal validity of the study.

#### Inclusion criteria

1. Being actively employed at the hospital during the study period
2. Voluntarily agreeing to participate in the study
3. Working in a nursing position
4. Not being on leave or medical report during data collection

#### Exclusion criteria

1. Refusing to participate
2. Working in a non-nursing position
3. Holding a managerial position (e.g. head nurse, charge nurse)

### Data collection procedure

Data were collected between April and June 2024. The instruments were administered offline (paper-based questionnaires) to accommodate nurses’ day and night shift schedules. Completed questionnaires were returned in sealed envelopes, with no identifying information collected, and confidentiality was assured for all participants. No monetary or non-monetary incentives were offered, and participation was entirely voluntary. The average response time was approximately 15–20 min.

Data collectors were individuals with research experience in nursing and were trained to ensure neutrality and consistency during administration. To minimize social desirability bias, anonymity was ensured, and all questions were phrased clearly, concisely, and in a manner that was easily understood. Data collection was scheduled during low workload periods (e.g. lunch hours and shift changes) to minimize fatigue and attention-related errors.

Before the main study, a pilot test was conducted with 15 nurses to assess the clarity, linguistic accuracy, and timing of the instruments. The pilot data were excluded from the final analyses.

*Variables:* The study's dependent variable was the level of organizational commitment of nurses, and the independent variable was the level of organizational blindness of nurses. Secondary variables were socio-demographic characteristics and working conditions. The study refrained from intervening to alter the effect of these variables on the dependent variable. The study examined the relationship between the organizational commitment scale and its sub-dimension scores, as well as the organizational blindness scale scores, among nurses working in the hospital.

*Data sources/measurement:* The present study gathered data through the Information Form, the Organizational Commitment Scale, and the Organizational Blindness Scale, all of which were developed by the researchers based on a comprehensive literature review.

### Information form

This form consists of 8 questions questioning the nurses’ socio-demographic characteristics and professional characteristics to be included in the study (Elibol *et al*., 2024; Şeker and Torun, 2021; Sarıköse *et al*., 2020).

### Organizational commitment scale

The Organizational Commitment Scale, developed by Meyer and Allen and adapted into Turkish by Wasti (2000), consists of three sub-dimensions: affective commitment, continuance commitment, and normative commitment. The affective commitment sub-dimension reflects an employee’s emotional attachment to, identification with, and involvement in the organization; the continuance commitment sub-dimension captures the perceived costs of leaving the organization; and the normative commitment sub-dimension reflects a sense of obligation to remain with the organization. Each item is rated on a five-point Likert scale, with higher scores indicating more substantial commitment. In the present study, the overall organizational commitment score was 3.59 ± 0.95, with sub-dimension means of 3.59 ± 1.34 for affective commitment, 3.67 ± 1.02 for continuance commitment, and 3.51 ± 1.15 for normative commitment. Although Cronbach's alpha values above 0.70 are generally considered indicative of good internal consistency, lower values (e.g. between 0.50–0.60) can be acceptable in certain circumstances—particularly in exploratory research, newly adapted scales, or subscales with fewer items. The continuance commitment sub-dimension includes only six items. As Cortina (1993)notes, alpha is sensitive to the number of items, with shorter scales often yielding lower coefficients despite conceptual validity. In line with this, Wasti's (2000) original Turkish adaptation reported a Cronbach's alpha of 0.58 for this sub-dimension. Therefore, the value observed in the current study (0.52) is consistent with previous research in similar contexts. Importantly, the items in this subscale measure a theoretically coherent construct, and retaining it is supported by prior studies that have used and validated the scale in the Turkish population.

### Organizational blindness scale

The Organizational Blindness Scale, developed by Seymen *et al*. (2016), consists of four dimensions: individual factors, work routine level, organizational structure, and sector structure, comprising a total of 24 items. Responses are measured on a five-point Likert-type scale, ranging from 1 = Strongly Disagree to 5 = Strongly Agree. The maximum possible score is 120, with score ranges classified as follows: 24–48 (low level), 49–84 (moderate level), and 85–120 (high level) of organizational blindness. Seymen *et al*. (2016) reported a Cronbach's alpha of 0.87 for the overall scale. In the present study, Cronbach’s alpha was found to be 0.848, indicating high internal consistency.

*Bias:* Several measures were implemented to minimize bias during data collection from nurses in the hospital. Anonymity and confidentiality were maintained to reduce social desirability bias, and the data collection process was standardized for all participants. Data collectors were trained to maintain impartiality, and the validity and reliability of the instruments used were ensured. The questions were designed to be clear and comprehensible. Data were collected during periods of lower workload to minimize errors due to inattentiveness. Participation was entirely voluntary, and all participants received clear and transparent information. A pilot study was conducted to assess the appropriateness of the methods, with independent researchers involved in the analysis process. The study's limitations were explicitly stated. These measures effectively minimized the risk of bias and enhanced the accuracy of the data collection process.

### Data analysis

Data were analyzed using IBM SPSS Statistics version 29.0. Descriptive statistics (mean, standard deviation, frequency, and percentage) were used to summarize demographic and study variables. Before inferential analyses, data normality was examined through skewness–kurtosis coefficients and visual inspection of histograms. The absence of outliers was confirmed using standardized residuals (±3.30) and Cook's Distance (<1.0). The Durbin–Watson value (1.46) indicated independence of residuals, and the Breusch–Pagan test (*p* = 0.399) verified homoskedasticity, confirming the suitability of the regression model.

Bivariate relationships between organizational blindness and the three dimensions of organizational commitment were evaluated using Pearson's correlation coefficients. To control for multiple comparisons, Holm–Bonferroni and Benjamini–Hochberg False Discovery Rate (FDR) corrections were applied where appropriate.

To identify predictors of organizational commitment, a simple linear regression analysis was first performed. Additionally, a binary logistic regression model was employed to examine how sociodemographic variables (age, gender, marital status, years of experience, and work unit) predicted high versus low levels of organizational commitment. Model fit was assessed using the Omnibus test (*χ*^2^ = 26.12, *p* = 0.006) and the Hosmer–Lemeshow test (*p* = 0.528), indicating good model adequacy. The Cox and Snell *R*^2^ (0.093) and Nagelkerke *R*^2^ (0.124) values were reported as indicators of explained variance.

All analyses were conducted at a 95% confidence level, and statistical significance was defined as *p* < 0.05 (two-tailed). Effect sizes were reported as Cohen's d for *t*-tests, partial η^2^ for ANOVA, and odds ratios (OR) with 95% confidence intervals for logistic regression outcomes.

### Ethical issues

This study adhered to the principles outlined in the Declaration of Helsinki (Brazil revision, 2013). Ethical approval for all procedures involving human participants was obtained from the Ethics Committee of a University in İstanbul (Decision number and date: 173/February 07, 2023) and institutional and study-specific permissions from the Ethics Committee of the Hospital (Decision number and date:255/December 13, 2023; 683/11 December 2023) before data collection. Informed consent was secured through a detailed process that provided participants with comprehensive information regarding the study's purpose, potential risks, benefits, and rights. Participation was voluntary, with participants retaining the right to withdraw at any stage of the study. The questionnaires were designed to maintain complete anonymity, and the data collected were utilized exclusively for this research. Strict measures were implemented to safeguard personal data, which was securely stored under the direct supervision of the research team. Additionally, written permissions were obtained from the respective scale developers.

This study was conducted as part of the master’s thesis of *Seda Berkay*, supervised by *Assistant Professor Dr Seda Tuğba Baykara Mat*, within the Master of Science in Nursing program. The supervisor contributed to the study design, methodological guidance, and interpretation of the results.

## Findings

Of the 280 nurses invited to participate in the study, 10 were on annual leave, resulting in an initial response rate of 96.6%. One questionnaire (0.36%) containing multiple responses was excluded from the dataset. Consequently, valid data were obtained from 269 nurses, corresponding to an adjusted response rate of 96.7%.

### Defining characteristics

This section presents the findings obtained from the questionnaires completed by the nurses participating in the study. As shown in Table 1, 54.3% of the participants fell within the age range of 26–35, 83.6% were female, 51.3% were married, and 66.5% held an undergraduate degree.

**Table 1:** Distribution of socio-demographic characteristics of nurses (*N* = 269)

Değişkenler	*n*	%
*Age groups*		
18–25 Years	58	21.6
26–35 Years	146	*54.3*
Over 36 Years	65	24.2
*Gender*		
Female	225	*83.6*
Male	44	16.4
*Marital status*		
Married	138	*51.3*
Single	131	48.7
*Education level*		
High school	31	11.5
Associate degree	22	8.2
Bachelor's degree	179	*66.5*
Postgraduate	37	13.8

**Figure 1:**
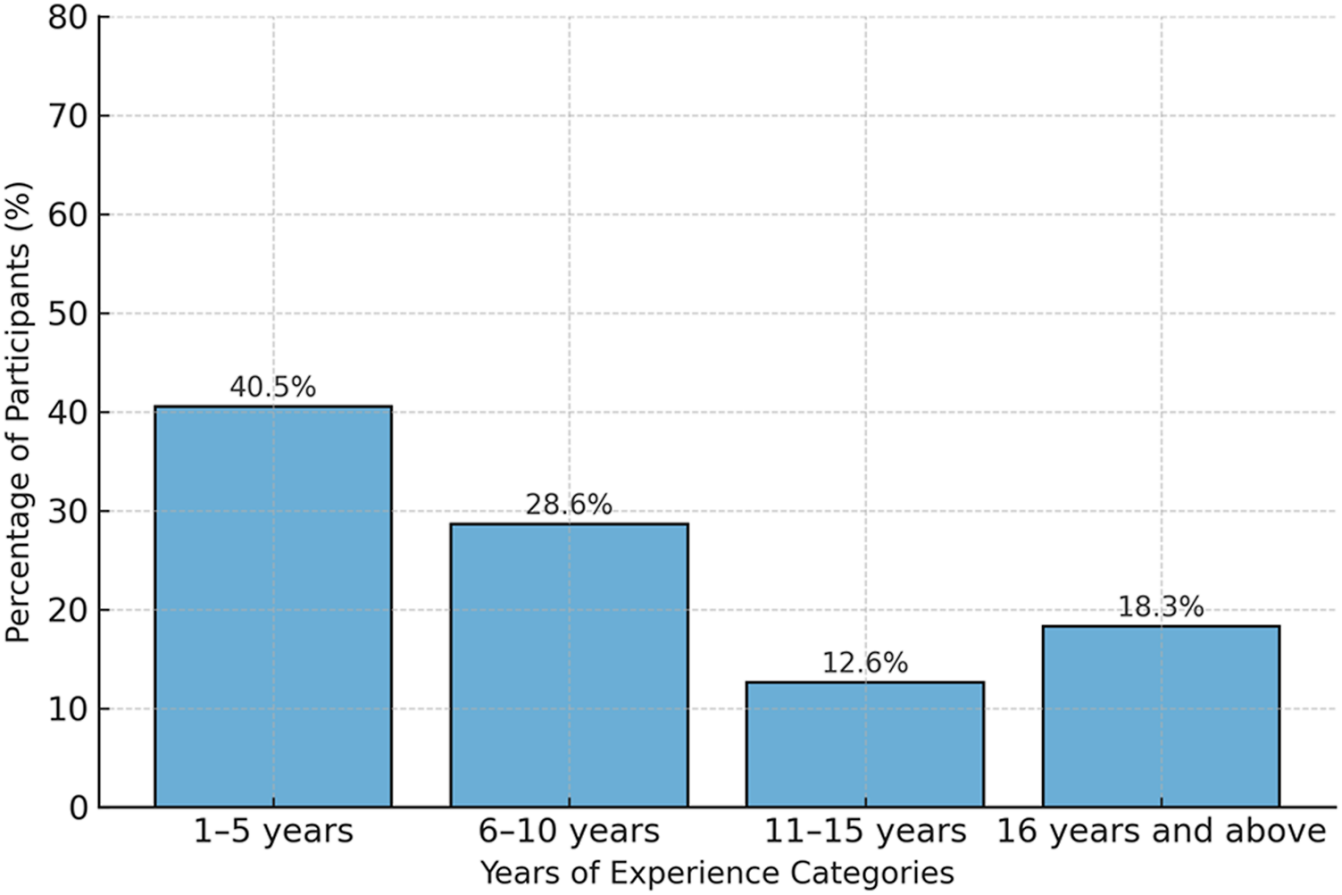
Distribution of participants by years of experience (%)

**Figure 2:**
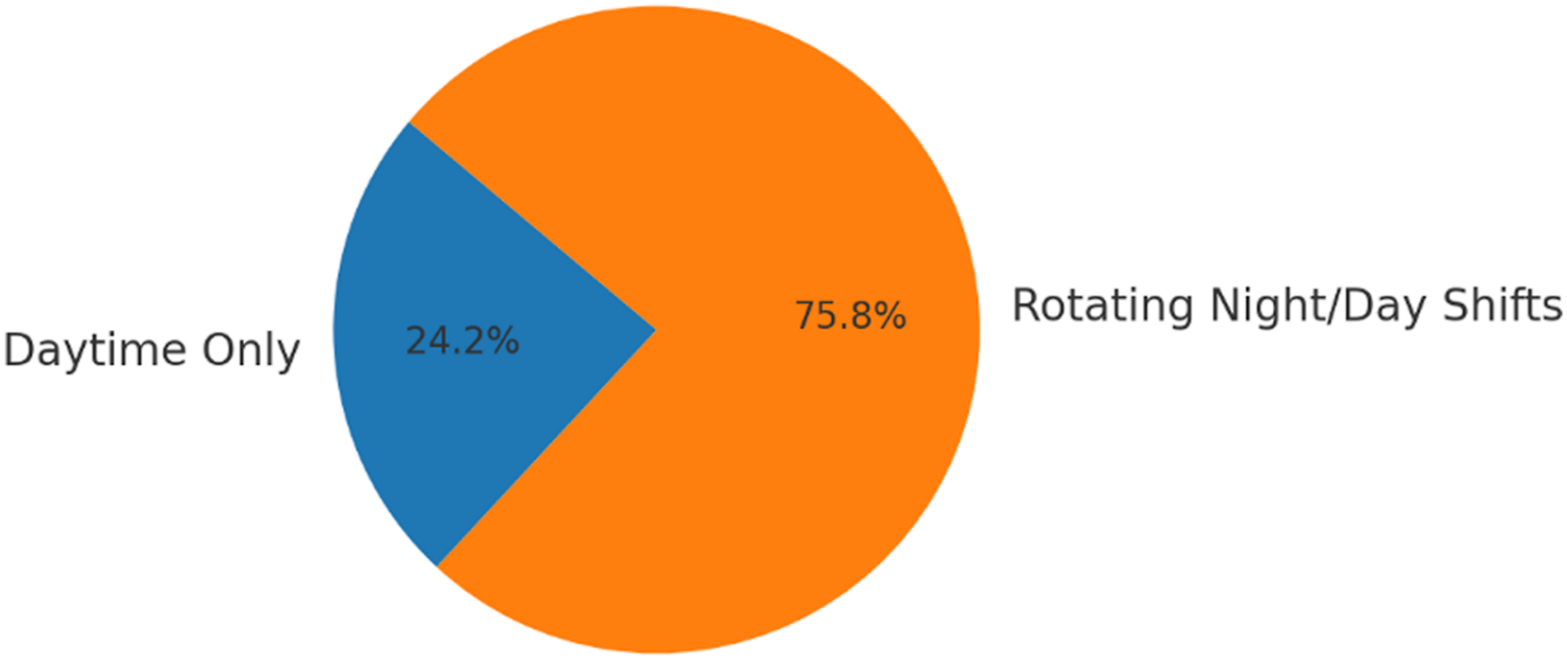
Distribution of participants by working schedule (%)

**Figure 3:**
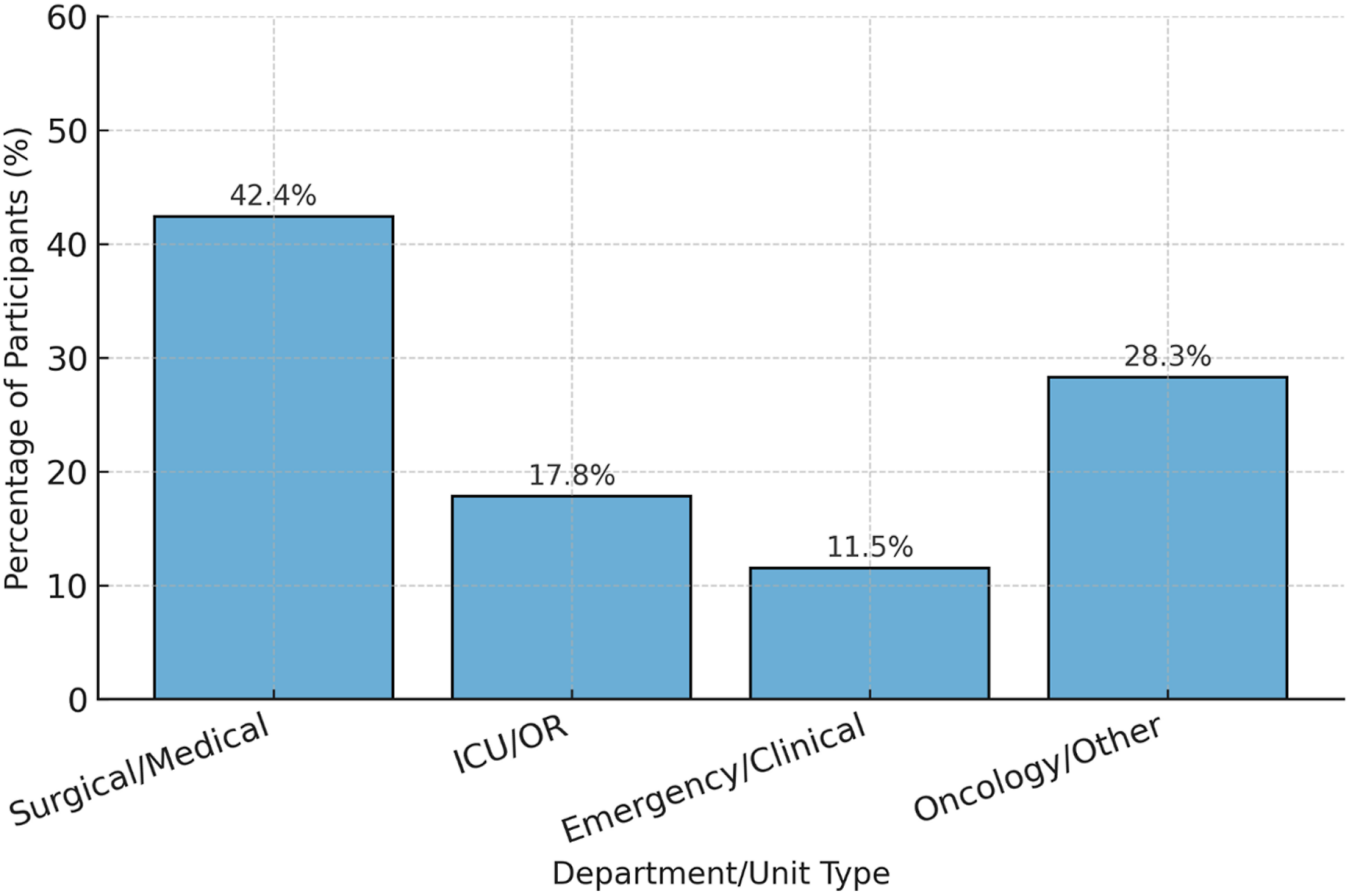
Distribution of participants by unit of employment (%)

**Figure 4:**
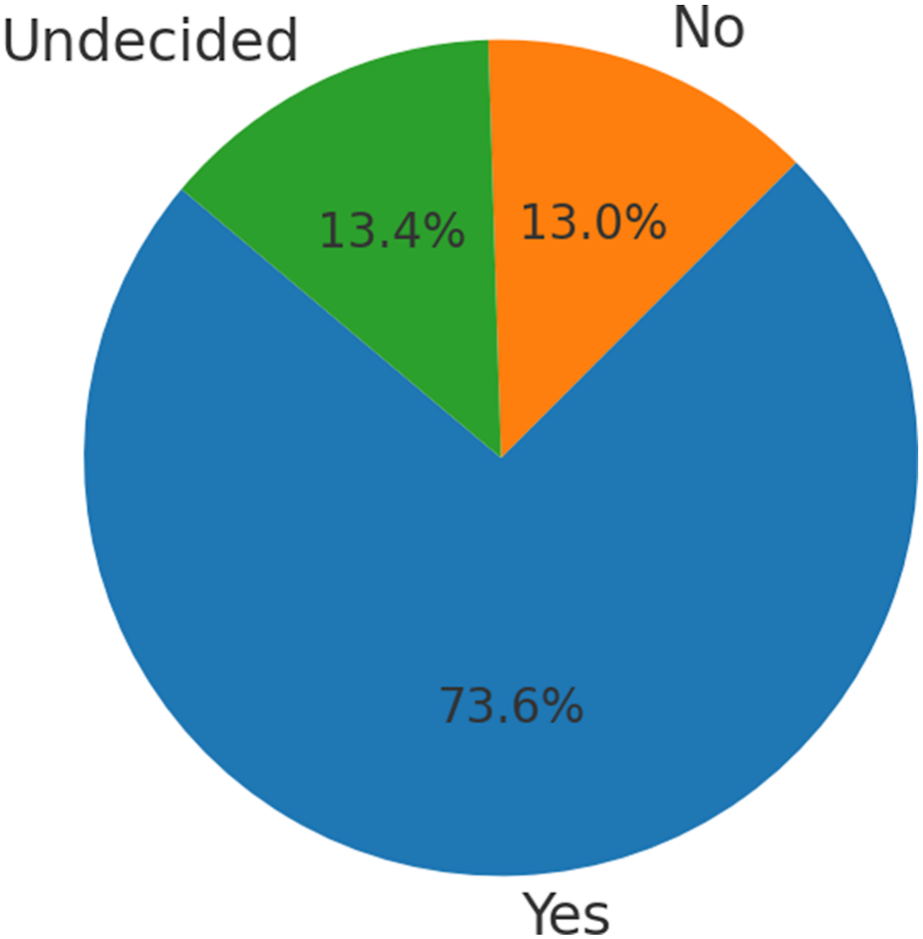
Intention to choose nursing again (%)

According to Figure 1, 40.5% of the nurses had 1–5 years of professional experience. Regarding their work schedules (Figure 2), 75.8% of the participants reported working alternating day and night shifts. As illustrated in Figure 3, 42.4% were employed in surgical or internal medicine clinics. Furthermore, as shown in Figure 4, 73.6% of the nurses stated that they would choose the nursing profession again.

Table 2 presents the comparison of nurses’ organizational commitment and organizational blindness scores across demographic and professional characteristics.

**Table 2:** Comparison results of scale scores of nurses

Variables	*n*	Organizational commitment scale		Affective commitment AB		Continuance commitment AB		Normative commitment AB		Organizational blindness scale	
		Mean	Sd	Mean	Sd	Mean	Sd	Mean	Sd	Mean	Sd
*Age groups*											
a-18–25 Years	*58*	3.65	0.80	3.87	0.98	3.52	0.92	3.56	1.18	57.62	10.23
b-26–35 Years	*146*	3.42	0.98	3.34	1.43	3.61	1.02	3.30	1.13	57.76	13.00
c-Over 36 Years	*65*	3.94	0.93	3.91	1.28	3.97	1.05	3.95	1.05	59.85	13.25
F/p		F: 7.26 *p:.001 c > b*		F: 5.81 *p:.003 a>b-c > b*		F: 3.75 *p:.025 c>a-c > b*		F: 7.53 *p: 0.001 c > b*		F:.713 *p*:.491 –	
*Gender*											
Female	*225*	3.62	0.99	3.62	1.38	3.74	1.02	3.51	1.18	*57.14*	*12.27*
Male	*44*	3.44	0.74	3.47	1.09	3.33	0.95	3.54	1.01	*63.82*	*12.34*
t/p		*t*:1.14 *p*:.255		*t*:.695 *p*:.487		*t*:2.50 *p:.013*		*t*: 0.169 *p*:.866		*t*: 3.29 *p:.001*	
*Marital status*											
Married	*138*	3.85	0.94	3.88	1.32	3.92	0.97	3.75	1.13	*57.07*	*12.79*
Single	*131*	3.33	0.90	3.30	1.30	3.42	1.01	3.26	1.12	*59.47*	*12.13*
t/p		*t:4.65 p:.000*		*t:3.62 p:.000*		*t:4.11 p:.000*		*t:3.55 p:.000*		*t: 1.57 p:.116*	
*Education level*											
High School	*31*	3.81	0.65	3.90	1.04	3.76	1.04	3.78	0.83	*56.48*	*15.15*
Associate Degree	*201*	3.55	0.95	3.54	1.36	3.62	0.99	3.50	1.16	*58.34*	*12.49*
Bachelor's Degree	*37*	3.64	1.16	3.62	1.45	3.92	1.15	3.39	1.30	*59.14*	*10.16*
F/p		F: 1.06 *p*:.345		F: 0.983 *p*:.376/---		F: 1.54 *p*:.215/---		F:.953 *p*:.387/---		F:.405 *p*:.668/---	
*Years of work experience*											
a-1-5 Years	*109*	3.44	0.94	3.43	1.35	3.51	1.02	3.37	1.18	*59.17*	*12.36*
b-6-10 Years	*77*	3.43	0.93	3.46	1.34	3.55	0.92	3.29	1.11	*55.64*	*12.47*
c-11–15 Years	*34*	3.74	0.88	3.79	1.24	3.88	1.08	3.56	1.03	*60.03*	*12.48*
d-Over 16 Years	*49*	4.09	0.90	4.03	1.28	4.10	1.01	4.15	1.02	*58.98*	*12.68*
F/p		F:6.89 *p:.000* d>a – d > b		F:2.82 *p:.039* d>a – d > b		F:4.79 *p:.003* d>a – d > b		F:7.02 *p:.000* d>a – d > b – d > c		F:1.61 *p*:.186	
*Work schedule*											
Daytime Only	*65*	3.78	1.08	3.97	1.39	3.71	1.19	3.66	1.33	*55.43*	*12.89*
Rotating Night/Day Shifts	*204*	3.54	0.90	3.48	1.30	3.66	0.96	3.47	1.09	*59.13*	*12.28*
t/p		*t:*1.80 *p:.072*		*t:2.60 p:.010*		*t:.343 p:*.732		*t:*1.16 *p:*.246		*t:* 2.08 *p:.038*	
*Work unit*											
a-Surgical/Medical Clinics	*114*	3.51	0.93	3.44	1.32	3.77	0.99	3.32	1.08	*59.52*	*10.81*
b- Intensive Care Units (GICU/CVICU) or Operating Room	*48*	3.52	0.95	3.53	1.40	3.55	1.03	3.48	1.14	*59.75*	*13.06*
c- Emergency or Clinic	*31*	3.75	0.76	3.81	1.20	3.77	1.14	3.67	0.93	*59.29*	*16.62*
d-Other (Chemotherapy/Oncology)	*76*	3.71	1.06	3.78	1.37	3.58	1.01	3.78	1.30	*54.92*	*12.24*
F/p		F:1.07 *p*:.361/---		F:1.30 *p*:.274/---		F:.861 *p*:.462/---		*F:2.96 p:.046* *d>a*		*F:2.529 p:.058*/---	
*Choosing the nursing profession again*											
a-Yes	*198*	3.70	0.96	3.78	1.31	3.74	0.98	3.58	1.18	*57.17*	*12.14*
b-No	*35*	3.44	0.74	3.28	1.32	3.63	1.00	3.42	0.89	*60.77*	*15.45*
c-Undecided	*36*	3.18	0.98	2.90	1.26	3.38	1.20	3.25	1.20	*61.64*	*10.54*
F/p		*F:5.24 p:.006 a>d*		*F:8.02 p:.000 a>b – a>c*		F:1.98 *p*:.140/---		F:1.37 *p*:.254/---		F:2.81 *p*:.062/---	

**Note(s):** F: One-Way Analysis of Variance – *t*: Independent Group *t*-Test, Mean: Arithmetic Mean, SD: Standard Deviation, SD: Standard Deviation, AB: Sub Dimension

A significant difference was found in overall organizational commitment by age group (*F* = 7.26, *p* = 0.001, η^2^ = 0.052), with nurses aged ≥36 years reporting higher scores than those aged 26–35. Significant but small differences were also found across affective, continuance, and normative subscales (η^2^ = 0.042, 0.027, and 0.054), with older nurses showing higher levels of all three commitment types.

Gender differences were observed in continuance commitment (*t* = 2.50, *p* = 0.013, d = 0.41), where female nurses scored higher, whereas male nurses exhibited higher organizational blindness (*t* = −3.29, *p* = 0.001, d = 0.53). Regarding marital status, married nurses showed significantly greater overall commitment (*t* = 4.65, *p* < 0.001, d = 0.56) and higher scores across all subscales. Work experience had a medium effect (*F* = 6.89, *p* < 0.001, η^2^ = 0.072); nurses with ≥16 years of experience reported higher commitment than those with fewer years in practice. Shift type also influenced results: nurses working daytime shifts had higher affective commitment (*t* = 2.60, *p* = 0.010, d = 0.36), while those on rotating shifts demonstrated higher organizational blindness (*t* = −2.08, *p* = 0.038, d = 0.29). Differences in normative commitment were observed across work units (*F* = 2.96, *p* = 0.046, η^2^ = 0.030), with oncology-unit nurses scoring higher than those in surgical or medical clinics. Finally, nurses who indicated they would choose the nursing profession again had significantly higher overall (*F* = 5.24, *p* = 0.006, η^2^ = 0.038) and affective commitment scores compared with those who were undecided or would not choose it again.

Overall, these results suggest that greater age, experience, marital stability, and consistent daytime work schedules are associated with stronger organizational commitment, whereas rotating shifts and male gender are linked to higher organizational blindness.

As shown in Table 3, there is a weak but statistically significant negative correlation between the overall scores of the Organizational Blindness Scale and the Organizational Commitment Scale (r = −0.266, *p* < 0.001). In addition, a weak negative association was found between organizational blindness scores and normative commitment subscale scores (r = −0.167, *p* < 0.05). However, a moderate negative correlation was observed between organizational blindness and affective commitment (r = −0.376, *p* < 0.001).

**Table 3:** Correlation coefficient results of organizational commitment scale scores and organizational blindness scale scores

Variables	Organizational commitment	Affective commitment	Continuance commitment	Normative commitment	Organizational blindness
Organizational Commitment	*1*	*-*	*-*	*-*	*-*
Affective Commitment	***	*1*	*-*	*-*	*-*
Continuance Commitment	***	***	*1*	*-*	*--*
Normative Commitment	***	***	***	*1*	
Organizational Blindness	−2.66**	−0.376**	*−0.065*	−0.167**	*1*

**Note(s):** **: *p* < 0.001 – *: *p* < 0.05

As shown in Table 4, the logistic regression model significantly predicted nurses’ organizational commitment status (Omnibus *χ*^2^ = 26.12, df = 4, *p* = 0.006), confirming that the independent variables collectively explained a meaningful proportion of variance. The model demonstrated an acceptable fit (Hosmer–Lemeshow *χ*^2^ = 6.10, df = 7, *p* = 0.528) and a classification accuracy of 57.6%.

**Table 4:** Logistic regression analysis results for the organizational commitment scale groups (*n* = 269)

Variables	B	SE	Wald	df	*p*	Odds Exp(B)	95% confidence interval	
							Lower	Upper
*Model (Final)*								
*F1: Gender*	0.086	0.359	0.057	1	0.812	1.08	0.539	2.20
*F2: Age*			4.89	2	0.086			
*F3: Marital Status*	0.721	0.335	4.63	1	0.031	2.05	1.066	3.96
*F4: Years of Experience*			8.06	3	0.045			
*F4: Years of Experience (1)*	−2.19	0.849	6.66	1	0.010	0.112	0.021	0.590
*F4: Years of Experience (2)*	−1.77	0.814	4.74	1	0.029	0.170	0.034	0.836
*F4: Years of Experience (3)*	−1.50	0.608	6.13	1	0.013	0.221	0.067	0.730
*F5: Work Schedule*	−0.396	0.365	1.17	1	0.278	0.673	0.329	1.37
*F6: Work Unit*	−0.396	0.365	1.17	1	0.278	0.673	0.329	1.37
Constant	0.795	0.679	1.37	1	0.242	2.25		
*Model Fit Results*								
Omnibus Test of Model Coefficients: *χ*^2^ = 26.12, df = 4, *p* = 0.006								
Hosmer–Lemeshow Goodness-of-Fit Test: *χ*^2^ = 6.10, df = 7, *p* = 0.528								
Model Classification Accuracy: 50%								
Indicators of the Effect of Independent Variables on the Dependent Variable								
Cox and Snell *R*^2^ = 0.093 (9.3%) Nagelkerke *R*^2^ = 0.124 (12.4%)								

**Note(s):** Independent Variables: gender, age, marital status, and years of experience. Dependent Variable: Organizational Commitment Scale (0 = low, 1 = high) *p < 0.05

The model explained approximately 9–12% of the variance in organizational commitment (Cox and Snell *R*^2^ = 0.093; Nagelkerke *R*^2^ = 0.124). Among the predictors, marital status emerged as a significant variable (*p* = 0.031): married nurses were 2.05 times more likely to report higher commitment than single nurses (OR = 2.05, 95% CI [1.07–3.96]). Years of experience also significantly influenced commitment (*χ*^2^ = 8.06, df = 3, *p* = 0.045). Compared with nurses having 1–5 years of experience, those with 6–10 years (OR = 0.112, *p* = 0.010), 11–15 years (OR = 0.170, *p* = 0.029), and ≥16 years (OR = 0.221, *p* = 0.013) were statistically less likely to fall into the low commitment group, reflecting an overall trend of higher commitment with increasing tenure. Gender, age, work schedule, and unit did not significantly contribute to the model (*p* > 0.05). Collectively, these findings indicate that marital stability and professional experience play central roles in predicting nurses' organizational commitment, whereas other demographic variables exert minimal influence.

In summary, the model was overall significant, and the results demonstrated that being married and having longer years of professional experience significantly increased the likelihood of higher organizational commitment among nurses.

The classification results for organizational commitment status groups are presented in Table 5. As shown in the table, based on a cut-off value of 0.50, the model correctly classified 54.8% of participants with *low* organizational commitment and 60.1% of those with *high* commitment. The overall accuracy rate of 57.6% suggests that the model demonstrated a moderate level of predictive performance.

**Table 5:** Classification results for organizational commitment status groups

Observed	Predicted		Percentage correct
Organizational commitment status	Low	High	
Low	69	57	54.8
High	57	86	60.1
*Overall percentage*			57.6
*Cut-off value (critical threshold)*			0.50

Before performing the regression analyses, the normality assumptions of the scales were assessed. Potential outliers were also examined using standardized Z-scores, and no extreme values were detected. As shown in Table 6, the skewness and kurtosis values for both scales ranged between −1 and +1, indicating that the data were approximately normally distributed.

**Table 6:** Results of descriptive values ​​calculated for normality test of scales used in regression analysis

Measures	*N*	Mean	Median	Standard deviation	Skewness	Kurtosis	Range
Organizational Blindness Scale (Total)	269	58.23	58.00	12.50	−0.122	−0.112	63.00
Organizational Commitment Scale (Total)	269	3.67	3.59	0.952	−0.142	−0.481	6.06

**Figure 5:**
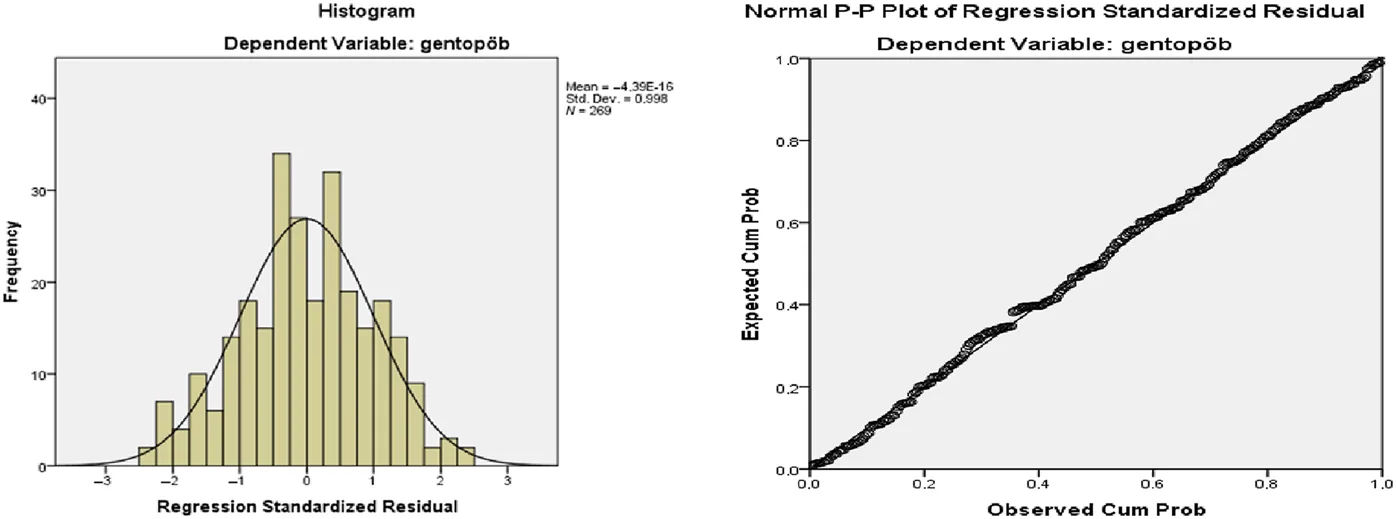
Scatter diagram results of the data used in regression analysis

The existence of a linear relationship among variables and the normal distribution of prediction errors were evaluated using a scatter plot. The scatter plot revealed a linear trend between the variables, confirming that the assumptions of linearity and normality of residuals, which are fundamental to regression analysis, were met (see Figure 5).

### Heteroskedasticity and homoskedasticity results

In linear regression analysis, homoskedasticity refers to the consistency of variance in the residuals. Verifying this assumption is essential to ensure the reliability and stability of regression estimates in both simple and multiple regression models. The assumption was tested using the Breusch–Pagan test, and the results are presented in Table 7. As shown in the table, the obtained *p*-value (*p* = 0.399) exceeded the 0.05 significance level, indicating that the variance of the residuals remained constant. This finding confirms that the assumption of homoskedasticity was met, meaning the residual variance did not vary significantly with the magnitude of the independent variable. Therefore, it can be concluded that no heteroskedasticity problem was present, and the regression results were statistically reliable.

**Table 7:** Results of the constancy of variance (Homoscedasticity) in the regression model according to the Breusch–Pagan test

	Model	Sum of squares	df	Mean square	F	p*
1	Regression	0.824	1	0.824	0.713	0.399^b^
	Residual	308.59	267	1.15		
	Total	309.41	268			

**Note(s):** a. Dependent Variable: res^2^ (squared residuals)

b. *Predictors: (*Constant*), organizational blindness (gentopok)*

*p* > 0.05

### Absence of outliers in the obtained data

To identify potential outliers, standardized residuals (Std. Residual) and Cook's Distance values were calculated. The results of this analysis are presented in Table 8. As shown in the table, standardized residuals are expected to fall within the range of −3.30 to +3.30, and the values obtained in this study were within these limits (min = −2.39; max = 2.49). Likewise, Cook's Distance values below 1.00 indicate the absence of influential outliers. The maximum Cook's Distance value observed (0.050) was well below this threshold. Therefore, it can be concluded that the dataset contained no outliers and that the regression model was free from influential observations.

**Table 8:** Results of standardized residual and Cook's distance values

Measures	Minimum	Maximum	Mean	Standard deviation	*n*
Standardized residual	−2.39	2.49	0.000	0.998	269
Cook's distance	0.000	0.050	0.004	0.006	269

### Independence of errors

The independence of errors was assessed using the Durbin–Watson statistic. A Durbin–Watson value between 1.0 and 3.0 indicates that the residuals are independent. The obtained value (DW = 1.46) was within this acceptable range, confirming that the residuals were independent and that autocorrelation was not present.

### Results of the regression analysis

The relationship between nurses’ organizational blindness and organizational commitment was examined using simple linear regression analysis. The results are presented in Table 9. As shown in the table, total scores on the *Organizational Blindness Scale* significantly predicted scores on the *Organizational Commitment Scale* (β = −0.266, *t* = −4.51, *p* < 0.001). The model explained 7.1% of the variance in organizational commitment (*R*^2^ = 0.071). The unstandardized regression coefficient (*B* = −0.020) indicated that a one-unit increase in organizational blindness was associated with a 0.020-point decrease in organizational commitment. These findings demonstrate that higher levels of organizational blindness were significantly associated with lower levels of commitment among nurses. The overall regression model was statistically significant (*F*(1, 267) = 20.38, *p* < 0.001), confirming that organizational blindness was a meaningful predictor of organizational commitment.

**Table 9:** Results of the standard regression analysis for predicting total scores of the organizational commitment scale (*n* = 269)

Variables	Unstandardized coefficients		Standardized coefficients	*t*	*p*	%95.0 confidence interval		Zero-order r	Partial r	Collinearity statistics	
	β	Standard error (SE)	Beta			Lower bound	Upper bound			*t*	VIF
Constant	4.775	0.268		17.84	0.000	4.249	5.302	–	–	–	–
Organizational blindness scale (Total)	−0.020	0.004	−0.266	−4.51	0.000	−0.029	−0.011	−0.266	−0.266	1.000	1.000
Model summary	R		*0.266*								
	*R* ^2^		*0.071*								
	Adjusted *R*^2^		*0.067*								
	F(1, 267) _(1–198)_		*20.38*								
	*p* <		*0.000*								

**Note(s):** Dependent Variable: Organizational Commitment Scale – *p < 0.05 - T: Tolerance Value – VIF: Variance Increase Factor R = 0.266 *R*^2^ = 0.071 Adjusted *R*^2^ = 0.067 *F*(1, 267) = 20.38 *p* < 0.001 Dependent Variable: Organizational Commitment Scale *p* < 0.05; T: Tolerance Value; VIF: Variance Increase Factor

## Discussion

This study is among the few to explore the link between organizational blindness and organizational commitment among nurses, providing early evidence that addressing these two constructs together is essential for building transparent, learning-oriented organizational cultures in healthcare. The results showed a statistically significant but weak negative correlation between blindness and commitment, suggesting that limited transparency, poor participation, and weak communication can erode nurses’ emotional and moral attachment to their organizations. Although the effect size was small, blindness still explained a meaningful portion of the variance, indicating that even subtle deficiencies in awareness and communication can weaken trust and engagement (Morrison and Milliken, 2000; Vakola, 2016; Grego-Planer, 2019; Herrera and De Las Heras-Rosas, 2021; Fronzetti Colladon *et al*., 2023).

Demographic and professional factors also shaped commitment levels: older (≥36), married, day-shift, and oncology-unit nurses demonstrated stronger commitment, consistent with prior studies showing that affective and normative commitment increase with age and experience (Arı *et al*., 2017; Güdük and Önder, 2021; Labrague *et al*., 2018). Married and long-tenured nurses’ sense of loyalty and moral obligation aligns with Meyer and Allen’s (1991) model, while higher blindness among male nurses may reflect gendered role expectations (Ghaibi *et al*., 2022). Continuance commitment emerged as the strongest subdimension—possibly reflecting career investments, financial stability, and job security—whereas lower normative commitment may be linked to limited advancement opportunities and insufficient leadership support (Elibol *et al*., 2024; Şeker and Torun, 2021).

Psychometric analyses revealed that the continuance commitment subscale had relatively low internal consistency, but additional diagnostics (item–total correlations, McDonald's ω, factor analyses) confirmed that its structure remained theoretically sound. This aligns with cross-cultural findings suggesting that continuance commitment is more context-sensitive and reflects pragmatic rather than emotional retention motives (Wasti, 2000; Neves *et al*., 2022).

The negative relationship between blindness and commitment likely reflects an erosion of psychological safety, trust, and perceived justice—conditions that arise when leaders overlook systemic problems or fail to engage with staff feedback. Over time, such dynamics reduce belonging and shared purpose, leading to emotional disengagement and weakened loyalty. To counter this, organizations must cultivate transparency, open dialog, and reflective leadership that actively renews awareness and learning rather than allowing hierarchical inertia to dominate.

Reducing organizational blindness requires system-wide strategies—not just individual awareness building. Supportive leadership, continuous learning, and open communication are vital foundations. Targeted interventions for younger, single, and shift-working nurses—such as mentorship and career development programs, fair scheduling, recognition systems, and equitable growth opportunities—can help strengthen their sense of belonging and reduce disengagement. Embedding transformational leadership and organizational learning principles can further promote psychological safety and encourage nurses to share concerns openly (Aydın and Sağır, 2021; Acar and Mete, 2023). Collectively, these measures can help healthcare institutions build more ethical, engaged, and resilient workforces, thereby enhancing both nurse well-being and patient safety.

## Conclusion

This study revealed a statistically significant but modest negative relationship between organizational blindness and nurses' organizational commitment. In other words, as blindness increases, nurses tend to feel less emotionally and morally attached to their organizations. Limited awareness, weak communication, and insufficient feedback systems within healthcare settings may gradually erode a sense of belonging and engagement among staff. Although the relationship was significant, the effect size was small suggesting that organizational commitment is a complex and multifaceted concept, influenced by many interrelated factors beyond blindness alone.

The findings also showed that marital status and professional experience play an important role. Married and long-tenured nurses were more likely to feel committed to their organizations, supporting Meyer and Allen's (1991) three-component model, especially in relation to normative commitment. Differences across shift types, unit assignments, and career choices indicated that stable work routines, continuous patient care (as in oncology or surgical wards), and intrinsic motivation all contribute to higher commitment levels. Together, these results emphasize that nurses’ organizational commitment is shaped by both relational and contextual factors within their working environments.

## Managerial implications

From a managerial perspective, these findings highlight the importance of leadership practices that simultaneously reduce organizational blindness and strengthen nurses' commitment. Hospitals should foster organizational awareness, transparent communication, and a culture of continuous learning to build trust and collaboration. Because blindness levels were higher among rotating and night-shift nurses, revising shift patterns and balancing workloads may help alleviate fatigue and disengagement. Married and long-tenured nurses—who demonstrated stronger commitment—can serve as mentors for younger or early-career staff, supported by structured mentorship and career development programs that enhance professional confidence and belonging. Implementing fair and flexible scheduling, recognition and reward systems, and clear career advancement opportunities can further increase motivation and organizational loyalty. To assess the effectiveness of these initiatives, hospitals should regularly monitor measurable indicators such as turnover rates, safety climate surveys, incident reports, and engagement scores. Ultimately, by encouraging transformational leadership and maintaining a psychologically safe culture where feedback and reflection are valued (Aydın and Sağır, 2021; Acar and Mete, 2023), healthcare organizations can build a more ethical, resilient, and committed nursing workforce—contributing to both employee well-being and the quality of patient care.

## Limitations and future research directions

This study was conducted in a single private hospital, where distinctive features such as 12-h shifts, lack of rotation, and performance-based management may limit generalizability. Although key regression assumptions were satisfied, the cross-sectional, single-site design restricts causal interpretation and temporal analysis. The Istanbul-based sample may also reflect cultural or institutional dynamics not representative of other healthcare settings. Additionally, the continuance commitment subscale showed relatively low reliability; however, supplementary analyses confirmed that the overall results remained consistent whether or not the subscale was included. Future studies should adopt multi-center and longitudinal designs to better understand cause-and-effect relationships.

## Practical and social impact

Improving nurses’ organizational commitment while reducing blindness has meaningful practical and social benefits. When nurses feel valued and connected to their organizations, they are more likely to stay in their jobs, experience greater satisfaction, and actively participate in quality and safety initiatives. Likewise, when organizational blindness decreases, hospitals can identify problems earlier, prevent adverse events, and strengthen a culture of safety and openness. Together, these improvements contribute to better patient outcomes, stronger institutional performance, and greater public trust in healthcare services.

## Data Availability

The datasets generated and/or analyzed during the current study are not publicly available due to institutional confidentiality policies and ethical restrictions. Still, they are available from the corresponding author upon reasonable request.
